# OncoTrace‐TOO: Interpretable Machine Learning Framework for Cancer Tissue‐of‐Origin Identification Using Transcriptomic Signatures

**DOI:** 10.1002/cnr2.70311

**Published:** 2025-08-10

**Authors:** Yang Hao, Haochun Huang, Daiyun Huang, Jianwen Ruan, Xin Liu, Jianquan Zhang

**Affiliations:** ^1^ Hepatobiliary and Pancreatic Surgery Central South University Xiangya School of Medicine Affiliated Haikou Hospital Haikou China; ^2^ School of Life Sciences and Technology Shanghai Jiao Tong University Shanghai China; ^3^ Wisdom Lake Academy of Pharmacy Xi'an Jiaotong‐Liverpool University Suzhou China; ^4^ Department of Biosciences and Bioinformatics Xi'an Jiaotong‐Liverpool University Suzhou China; ^5^ Hepatology Central South University Xiangya School of Medicine Affiliated Haikou Hospital Haikou China

**Keywords:** cancer of unknown primary, machine learning, metastasis, tissue‐of‐origin identification, transcriptomics

## Abstract

**Background:**

Cancer of unknown primary remains a formidable diagnostic challenge due to the inability to pinpoint the primary tumor site, which restricts the use of targeted therapeutics. Although machine‐learning methods that integrate transcriptomic approaches have provided valuable insights into tumor origins, they often face challenges in distinguishing biologically similar tumors and typically lack biological interpretability.

**Aims:**

This study aims to develop a transparent and biologically interpretable machine learning framework to accurately classify tissue‐of‐origin across diverse cancer types, thereby facilitation clinical diagnosis.

**Methods:**

We designed OncoTrace‐TOO, a novel tissue‐of‐origin classification model based on gene expression profiles. The model utilizes pan‐cancer discriminative molecular features identified through one‐vs‐rest differential expression analysis and applies logistic regression as the classification algorithm.

**Results:**

OncoTrace‐TOO achieved an overall accuracy of 0.967, with perfect classification for seven cancer types (e.g., CHOL, DLBC, and LAML). The model demonstrated high predictive accuracy in both primary and metastatic cancers across TCGA and GEO validation datasets, with enhanced capability in resolving histologically related malignancies as well as classifying rare cancer subtypes. When applied to independent clinical tumor samples, the model achieved TOO prediction accuracies of 0.857, further validating its robustness. Importantly, the framework offers biologically interpretable predictions by revealing tumor‐specific molecular signatures, thus enhancing its clinical applicability.

**Conclusions:**

OncoTrace‐TOO not only offers high predictive accuracy for tissue‐of‐origin classification, but also delivers biologically meaningful insights that support clinical decision‐making. This framework holds promise for improving diagnostic precision and guiding personalized treatment in challenging cancer cases.

## 1 Introduction

Cancer of unknown primary (CUP) refers to metastatic tumors where the primary site of origin cannot be identified through conventional diagnostic methods, typically histopathological examination. CUP accounts for approximately 3%–5% of all cancers worldwide, and due to its inherently aggressive behavior and the lack of site‐specific treatment strategies [1], patients with CUP often have a poor prognosis [2, 3]. Accurately identifying the potential primary site of CUP tumors and demonstrating clinical benefits from targeted therapies could substantially expand the available treatment options for these patients [4].

Pathological evaluation plays a critical role in determining the primary cancer type, relying on immunohistochemistry (IHC) results, tumor morphology, and clinical findings. However, in metastatic and poorly differentiated cancers, tumor morphology and immune response patterns may undergo significant changes due to metastasis and dedifferentiation, leading to a marked decline in the sensitivity and accuracy of these methods [5]. In CUP patients, IHC results indicative of a single primary diagnosis are found in only 25% of cases [6]. Furthermore, diagnostic imaging techniques, which rely on visible features to identify the primary tumor, are often limited and may be affected by artifacts [7, 8], with an accuracy rate of just 20% in diagnosing CUP.

Molecular tumor profiling has emerged as a promising alternative for primary site classification. This approach takes advantage of the quantitative nature of molecular features and demonstrates high accuracy in tumors of known cancer types. Given that metastatic tumors may retain the gene expression patterns specific to their primary tumor type, molecular classifiers based on the features offer significant potential for aiding diagnosis by identifying molecular pathological alterations [9]. Commercial assays like CancerTYPE ID, which utilize RT‐PCR profiling of 92 genes, have demonstrated approximately 85% accuracy across diverse malignancies. However, these tools relatively high costs and reduced performance in poorly differentiated or rare tumors have limited their widespread clinical applicability. Recent efforts have extended expression‐based classification approaches using transcriptome‐wide data derived from microarrays or RNA‐seq. For instance, TOD‐CUP [10], based on mRNA expression profiles, has demonstrated multi‐platform effectiveness but exhibits diminished performance when distinguishing tumors originating from anatomically or biologically similar tissues. Likewise, miRNA‐based [11] classifiers have been developed to improve the identification of poorly differentiated malignancies; however, the incomplete understanding of miRNA function undermines both their biological interpretability and translational potential. More recently, some AI‐driven tools like CUP‐AI‐DX [12], excel at extracting high‐dimensional features to identify tumor subtypes but often suffer from limited interpretability and poor generalization to rare cancers.

Moreover, existing computational approaches face two persistent trade‐offs: (1) high‐dimensional feature spaces increase the risk of overfitting [13] and reduce robustness to external datasets; (2) overly simplified gene panels or filters reduce model accuracy and fail to capture nuanced inter‐tumor heterogeneity. To mitigate these challenges, various statistical approaches—such as median absolute deviation (MAD) [10], relative expression ordering (REO) [14, 15], and correlation‐based [16] scoring have been proposed to enhance feature selection. Yet, finding an optimal balance between signal retention and model generalizability remains an unresolved problem.

In this study, we introduce OncoTrace‐TOO, a transparent and interpretable machine learning framework for tumor classification based on transcriptomic profiles. The model integrates an optimized one‐vs‐rest differential expression strategy with logistic regression to derive tissue‐specific gene signatures, enabling biologically grounded and reproducible predictions. Our results demonstrate that OncoTrace‐TOO achieved high classification accuracy across both primary and metastatic tumors in TCGA and GEO datasets (above 96.7%); it generalized robustly to independent prospective FFPE clinical samples (85.7% accuracy, excluding CUP cases). Notably, OncoTrace‐TOO shows marked improvements in tumor types that have been historically difficult to classify, highlighting its potential for use in diagnostically ambiguous cases. Beyond accurate classification, the model reveals gene‐level feature contributions, enabling clinicians to assess the biological plausibility of predictions and facilitating downstream experimental or pathological validation. These attributes collectively position OncoTrace‐TOO as a clinically actionable framework with the potential to advance molecular diagnosis and guide personalized treatment strategies in metastatic cancers, including CUP.

## 2 Methods and Materials

### 2.1 Datasets

#### 2.1.1 Model Training and Internal Validation Datasets

**FIGURE 1:**
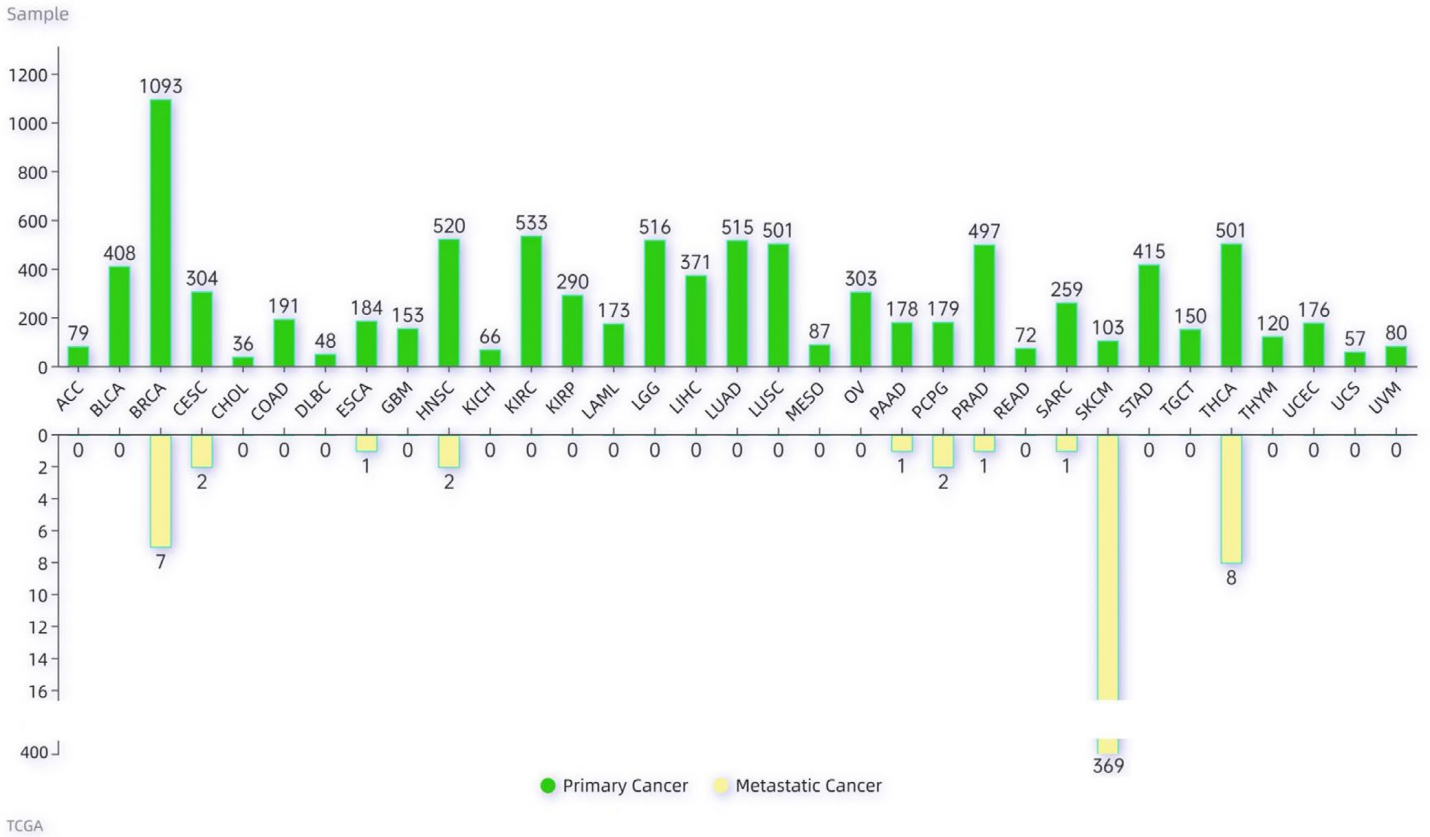
Distribution of TCGA primary and metastatic tumor samples across cancer types.

The normalized expression data on 20 501 unique genes for 33 cancer types was downloaded from The Cancer Genome Atlas (TCGA) and used to train the classifier. These cancer types include: Adrenocortical carcinoma (ACC), Bladder Urothelial Carcinoma (BLCA), Breast invasive carcinoma (BRCA), Cervical squamous cell carcinoma and endocervical adenocarcinoma (CESC), Cholangiocarcinoma (CHOL), Colon adenocarcinoma (COAD), Lymphoid Neoplasm Diffuse Large B‐cell Lymphoma (DLBC), Esophageal carcinoma (ESCA), Glioblastoma multiforme (GBM), Head and Neck squamous cell carcinoma (HNSC), Kidney Chromophobe (KICH), Kidney renal clear cell carcinoma (KIRC), Kidney renal papillary cell carcinoma (KIRP), Acute Myeloid Leukemia (LAML), Brain Lower Grade Glioma (LGG), Liver hepatocellular carcinoma (LIHC), Lung adenocarcinoma (LUAD), Lung squamous cell carcinoma (LUSC), Mesothelioma (MESO), Ovarian serous cystadenocarcinoma (OV), Pancreatic adenocarcinoma (PAAD), Pheochromocytoma and Paraganglioma (PCPG), Prostate adenocarcinoma (PRAD), Rectum adenocarcinoma (READ), Sarcoma (SARC), Skin Cutaneous Melanoma (SKCM), Stomach adenocarcinoma (STAD), Testicular Germ Cell Tumors (TGCT), Thyroid carcinoma (THCA), Thymoma (THYM), Uterine Corpus Endometrial Carcinoma (UCEC), Uterine Carcinosarcoma (UCS), and Uveal Melanoma (UVM). The TCGA barcodes were used to classify samples into either primary tumor (values 01 or 03) or metastatic tumor (values 06 or 07). This resulted in a final dataset which consists of 9158 primary and 394 metastatic tumor samples, forming the foundation for our analysis (detailed statistics are summarized in Figure 1).

For model development and internal validation, we applied a five‐fold cross‐validation strategy within the TCGA dataset. This procedure was used to systematically determine the optimal number of top‐ranked features (*k*) and to select the best‐performing combination of classification algorithms.

#### 2.1.2 External Validation Datasets

To rigorously validate the performance and generalizability of our model, we incorporated multiple external cancer datasets from the Gene Expression Omnibus (GEO) (Table 1). These datasets were selected based on their diverse cancer types and experimental conditions, which allowed for a comprehensive evaluation of the model across different biological and clinical scenarios.

**TABLE 1:** Summary of GEO external validation datasets.

Dataset ID	Cancer type	Sample size	References
GSE69164	Hepatocellular Carcinoma	11	Zhang et al. [17]
GSE164862	Bladder Cancer	12	Grunewald et al. [18]
GSE58708	Breast Cancer, Liver Metastasis	6	McBryan et al. [19]
GSE215384	Breast Cancer, Liver Cancer	4	Li et al. [20]
GSE213857	Clear Cell Renal Cell Carcinoma	47	Agudelo et al. [21]

The first dataset from Zhang et al. [17] (GSE69164) explored the relationship between hepatocellular carcinoma and portal vein tumor thrombus using RNA sequencing data from 11 primary liver cancer samples. The second dataset from Grunewald et al. [18] (GSE164862) examined the potential enhancement of CAR‐T cell cytotoxicity by DNMT inhibition, using RNA sequencing data from 12 bladder cancer samples. McBryan et al. [19] (GSE58708) provided a dataset of RNA sequencing from three primary breast cancer samples and three liver metastases samples, which was used to study changes in metastatic gene expression post‐endocrine therapy. In addition, Li et al. [20] (GSE215384) provided RNA‐Seq and ChIP‐Seq data from breast and liver cancer samples to investigate estrogen receptor α targets, using two samples from each cancer type. Finally, Agudelo et al. [21] (GSE213857) offered RNA sequencing data from 47 clear cell renal cell carcinoma (ccRCC) patients, providing insight into the metabolic characteristics of a xenotransplantation model.

#### 2.1.3 Clinical Validation Data

The clinical validation cohort consisted of 14 formalin‐fixed, paraffin‐embedded (FFPE) tumor tissue samples obtained from patients diagnosed with six cancer types: breast cancer, lung cancer, sarcoma, thyroid cancer, gastric cancer, and rectal cancer. All specimens exhibited > 80% tumor cellularity and were accompanied by complete clinicopathological annotations. Total RNA was isolated using the RNeasy FFPE Kit (Qiagen) with DNase I treatment, followed by quality assessment via Agilent 2100 Bioanalyzer (RIN ≥ 7.0). Strand‐specific cDNA libraries were prepared using the TruSeq Stranded Total RNA Library Prep Kit (Illumina) and sequenced on NovaSeq 6000 (Illumina) with 150 bp paired‐end reads, achieving > 40 million reads per sample. Raw sequencing data were processed through FASTQC (v0.11.9) for quality control and aligned to the GRCh38 human reference genome using STAR (v2.7.9a) with two‐pass mode. Gene‐level quantification was performed via RSEM (v1.3.3) with GENCODE v32 annotations. Batch effects were mitigated using Combat‐Seq normalization. This study was approved by the Central South University Xiangya School of Medicine Affiliated Haikou Hospital, and patients provided written informed consent to have their information used in the study.

### 2.2 Data Preprocessing and Normalization

The RNA‐Seq by Expectation–Maximization (RSEM) method [22] was used for normalization to correct technical biases introduced during library preparation and sequencing, thereby enabling accurate gene expression comparisons across all samples. After normalization, a zero‐deletion step was applied, excluding genes with over 25% zero expression values across the combined dataset of all samples, ensuring the retention of genes with sufficient data for reliable analysis. Due to the highly skewed read count distribution, a log2 transformation was subsequently applied to stabilize variance and normalize the data, reducing the influence of outliers. Following these preprocessing steps, the remaining genes were intersected across the 33 cancer types, resulting in unified training matrices containing 13 740 genes, which were then used for model development.

### 2.3 Feature Selection

In this study, we implemented a ‘one‐versus‐rest’ differential analysis across multiple cancer types. The *DESeq2* package in R, which uses a negative binomial distribution and a shrinkage estimator for dispersion parameters, was employed to model count data and identify differentially expressed genes. The procedure begins by selecting a target cancer type, followed by a differential analysis comparing two matrices: one containing the expression data for the target cancer type and the other composed of data from the remaining cancer types. This comparison yields gene names, log2 fold changes, and associated *p* values. Genes are then ranked based on their log2 fold change values, and the top‐*k* genes are selected for further investigation.

**FIGURE 2:**
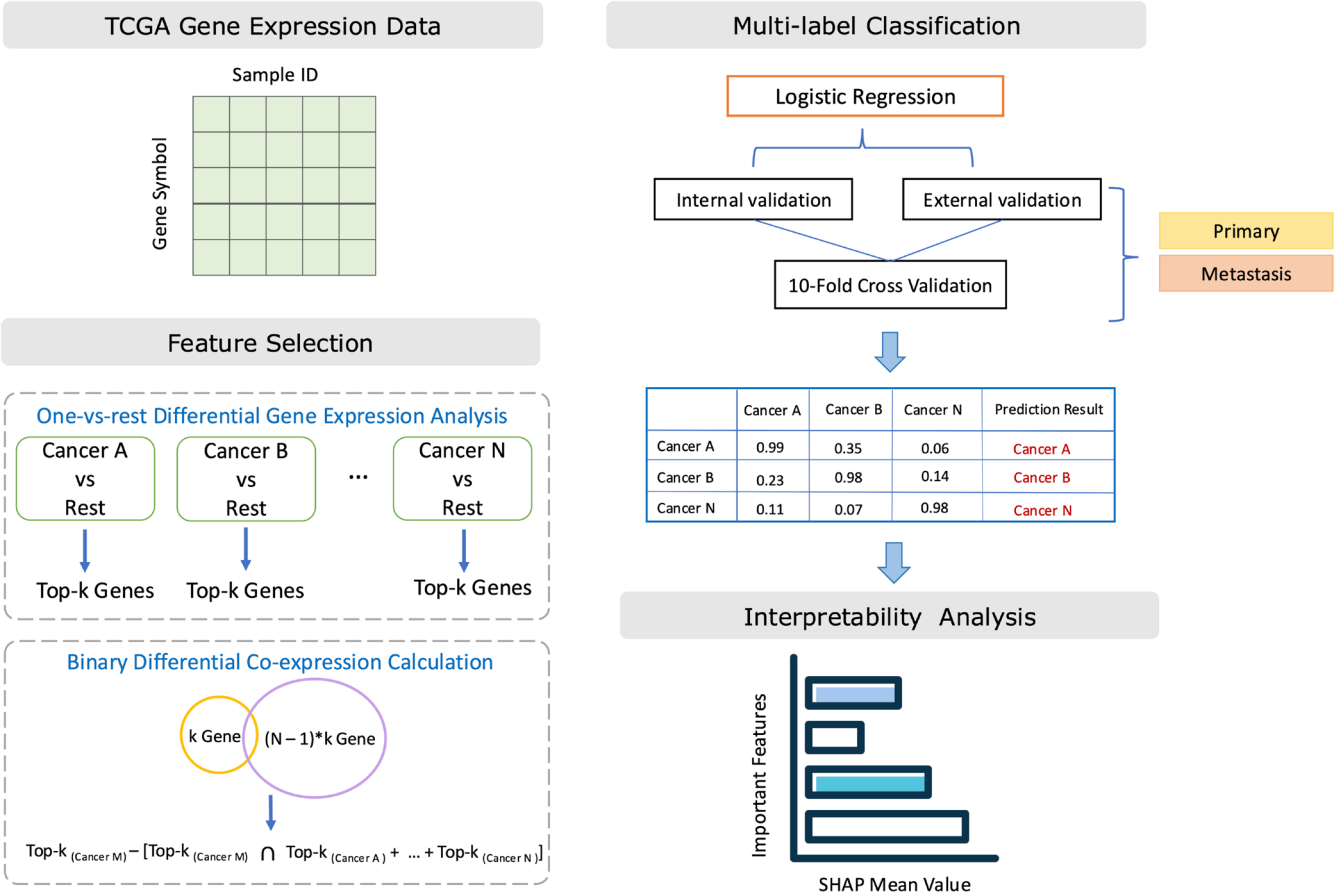
Workflow of OncoTrace‐TOO for TOO identification.

To refine the analysis, a specific target cancer type $S_{i}$ is chosen, and gene pairs from all other cancer types are aggregated into a secondary set $U_{j\neq i}S_{j}$. The difference $D_{i}$ between the gene sets of $S_{i}$ and $U_{j\neq i}S_{j}$ is then used to identify key markers specific to the target cancer type (Figure 2). The analysis was conducted for various values of top *k* (*k* = 50, 100, 150, 200, and 250), and corresponding result matrices for the identified genes were generated.

The one‐vs‐others difference set for a collection of sets $\left\{S_{1},S_{2},\ldots ,S_{n}\right\}$ can be written as:

(1) $$D_{i}=S_{i}-S_{i}\cap \left(U_{j\neq i}S_{j}\right)$$

Furthermore, the union of all such “one‐vs‐others” difference sets is given by:

(2) $$U=U_{i=1}^{n}D_{i}$$

From an initial pool of 20 501 genes, differential expression (DE) analysis efficiently selects a subset of *k* genes exhibiting the most significant statistical distinction between a given cancer type and all others. This targeted feature reduction is critical for mitigating the curse of dimensionality and minimizing overfitting in high‐throughput transcriptomic data.

### 2.4 Multitask Classifiers

#### 2.4.1 Logistic Regression

Logistic regression is a well‐established linear model commonly employed for classification tasks. In the multi‐class tumor classification problem, we extended the logistic regression model using the “one‐versus‐rest” (OvR) strategy. For each tumor type (class *S*_*i*_), a separate binary classifier was trained to distinguish whether a given sample belongs to that particular class. The final class assignment for each sample was based on the highest predicted probability from the individual classifiers. The model was trained using stochastic gradient descent (SGD) optimization, which iteratively updates the model parameters to minimize the loss function, ensuring efficient convergence to the optimal solution.

#### 2.4.2 Multilayer Perceptron

For the multilayer perceptron (MLP) model, gene expression data were first converted into tensor format using the PyTorch framework. The model architecture consisted of three fully connected layers: an input layer with 𝑁neurons, a hidden layer with 128 neurons, and an output layer with 33 neurons, corresponding to the number of tumor types. The hidden layer utilized the Sigmoid activation function to introduce non‐linearity, while the output layer applied the Softmax function to transform the logits into probability distributions over the classes. Model optimization was performed using cross‐entropy loss, with Stochastic Gradient Descent (SGD) as the optimization algorithm and a learning rate set to 0.001. To mitigate overfitting, L2 regularization (weight decay) was incorporated. The value of 𝑁 was determined based on the number of selected genes after applying each feature selection method. Each MLP model was trained for 10 000 epochs to ensure convergence.

Given the imbalance in the sample sizes, 10‐fold cross‐validation was employed to mitigate overfitting and selection bias. All models were implemented using the *scikit‐learn* package.

#### 2.4.3 Top Scoring Pairs

The top scoring pairs (TSP) classifier [23] was used to analyze gene expression profiles. The TSP algorithm operates by calculating a probability value $P_{\mathrm{ij}}\left(C\right)$ for each “marker gene pair” $\left(i,j\right)$, where C represents the class (1 or 2). This probability represents the likelihood that the expression value $V_{i}$ of gene i is smaller than the expression value $V_{j}$ of gene j for all samples in class C. The algorithm then computes the absolute score of the pair as:

(3) $$\Delta P_{\mathrm{ij}}=\left|P_{\mathrm{ij}}\left(1\right)-P_{\mathrm{ij}}\left(2\right)\right|$$

This score represents the information gain of the gene pair, quantifying its discriminative power between the two classes. Gene pairs are then sorted based on the $\Delta P_{\mathrm{ij}}$ score, and the top k pairs are selected.

For multiple categories (i.e., *C* > 2), a one‐vs‐one strategy is used, where each pairwise combination is tested to identify genes that are distinctive enough to differentiate between two classes. This approach is implemented using the R package ‘*MulticlassPairs*’ [24].

### 2.5 Evaluation Metrics

Each classification algorithm was initially assessed based on overall accuracy. Given the inherent class imbalance in cancer‐type distribution across datasets, we adopted class‐specific *F*1 scores as the principal performance metric. This approach aligns with established machine learning practices for imbalanced data, where *F*1 score (harmonic mean of precision and recall) provides more clinically actionable performance evaluation by jointly considering both false positives and false negatives in cancer‐type prediction.

$$\text{Accuracy}=\frac{\mathrm{TP}+\mathrm{TN}}{\mathrm{TP}+\mathrm{FN}+\mathrm{TN}+\mathrm{FP}}$$

$$\text{Recall}=\frac{\mathrm{TP}}{\mathrm{TP}+\mathrm{FN}}$$

$$\text{Precision}=\frac{\mathrm{TP}}{\mathrm{TP}+\mathrm{FP}}$$

$$F1=2\times \left(\text{Precision}\times \text{Recall}\right)/\left(\text{Precision}+\text{Recall}\right)$$

where TP, TN, FP, and FN represent true positives, true negatives, false positives, and false negatives.

### 2.6 Interpretability Analysis

SHAP provides feature attribution by calculating the contribution of each feature across all samples for various cancer types. To visualize the influence of the most important features on the model's predictions, we utilized a SHAP beeswarm plot. In these plots, the feature values are color‐coded: red for high values, blue for low values, and purple for intermediate values. The top 10 feature values are plotted along the x‐axis, illustrating the distribution of their impact on the model's output. To assess the global importance of each feature for a specific cancer type, we computed the average absolute SHAP value for each feature across samples associated with that cancer type. This process was repeated for all 33 cancer types, enabling us to evaluate the average contribution of each feature to the model's output for each type. This method allowed us to precisely assess the relevance of features in the context of different cancer types and identify cancer‐specific key genes. To further validate the selected features, we analyzed the top 10 most influential features for gynecological cancers and cholangiocarcinoma, focusing on their respective SHAP values. WebGestalt (https://www.webgestalt.org/) was used to identify the protein–protein interaction (PPI) subnetwork based on the key genes predicted by SHAP.

## 3 Results

### 3.1 Feature Selection and Model Optimization

To address the challenges of high dimensionality and overfitting inherent in transcriptomic data, we first implemented a one‐vs‐rest differential gene expression analysis to identify cancer‐specific biomarkers. Logistic Regression (LR) was selected as a representative machine learning (ML) method due to its ability to handle high‐dimensional datasets and provide interpretable variable importance analysis, which is instrumental in pinpointing key genes for tumor classification. In parallel, Multilayer Perceptron (MLP), a deep learning (DL) approach, was employed to capture non‐linear relationships in complex omics data, a capability critical for modeling intricate gene expression patterns.

**FIGURE 3:**
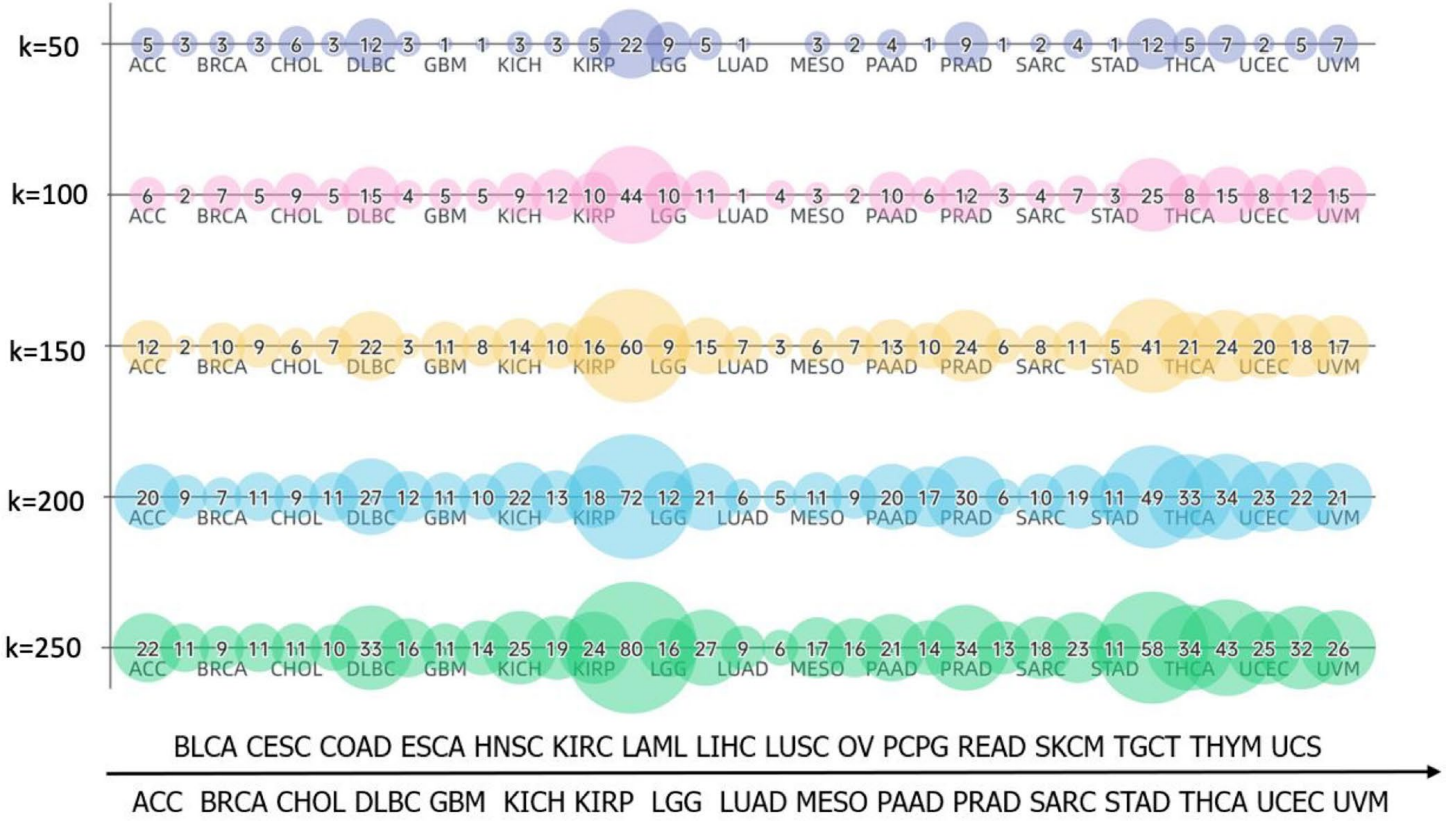
Distribution of genes across 33 cancer types identified by the “one‐versus‐rest” differential gene expression analysis with top‐*k* sizes of 50, 100, 150, 200, and 250.

Using these two frameworks, we systematically evaluated the impact of varying *k* values (50, 100, 150, 200, and 250) on classification accuracy across 33 cancer types. Our analysis revealed a strong correlation between the number of selected features and overall model performance (Figure 3). Specifically, the k threshold directly influenced the count of differentially expressed genes chosen for each cancer type. For instance, increasing *k* from 50 to 250 resulted in a progressive increase in the number of selected genes for LAML (22, 44, 60, 72, and 80 genes for *k* = 50, 100, 150, 200, and 250, respectively). Conversely, cancers such as LUAD and BLCA consistently exhibited fewer significant genes across all thresholds, reflecting the intrinsic biological variability in gene expression patterns for these cancer types. Notably, models trained with *k* = 200 achieved optimal performance, balancing feature richness and noise reduction. Both models using this threshold attained accuracies of 96.69% and 96.64%, respectively, on the TCGA test set (Table 2). Further increasing *k* to 250 did not improve accuracy (LR: 96.70%, MLP: 96.37%), confirming that excessive features introduced redundancy. Therefore, *k* = 200 was subsequently adopted for downstream comparative analyses.

**TABLE 2:** Performance comparison of MLP and LR classifiers with different gene selection strategies.

*k* value	Feature number	MLP (Accuracy %)	LR (Accuracy %)
*k* = 50	153	92.86	93.02
*k* = 100	297	95.67	94.98
*k* = 150	455	95.56	96.48
***k* = 200**	**611**	**96.64**	**96.69**
*k* = 250	739	96.37	96.70

*Note:* Bold values indicate the best *k* value.

### 3.2 Comparative Performance of OncoTrace‐TOO and Other Classifiers

To systematically evaluate the performance of OncoTrace‐TOO, we benchmarked five classification frameworks: logistic regression (LR), multilayer perceptron (MLP), XGBoost, CatBoost, and the k‐top scoring pairs (*k*‐TSP) method. As summarized in Table S1, OncoTrace‐TOO (LR with *k =* 200) achieved the highest accuracy (96.69%) and the lowest false‐negative rate (0.033) on the TCGA test set, outperforming both MLP (96.64%, 0.038) and ensemble methods like XGBoost (95.18%, 0.048). Critically, LR demonstrated a 15‐fold reduction in computational time compared to MLP (15 min vs. 90 min), highlighting its efficiency for large‐scale applications.

**FIGURE 4:**
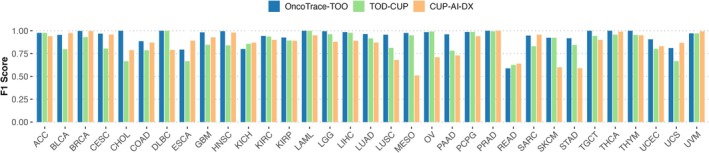
Performance comparison of OncoTrace‐TOO, TOD‐CUP, and CUP‐AI‐Dx for TOO classification across 33 cancer types.

We chose TOD‐CUP and CUP‐AI‐Dx, two representative transcriptome‐based methods as references, and compared their performance with OncoTrace‐TOO in terms of *F*1 score and overall accuracy. OncoTrace‐TOO exhibited marked superiority across all 33 cancer types, particularly in challenging cancer subtypes (Figure 4). Internal validation on TCGA data revealed near‐perfect classification (100% accuracy) for seven cancer types (e.g., CHOL, DLBC, LAML), with overall accuracy exceeding 96% across all 33 cancer types (Table 3).

**TABLE 3:** Overall accuracy comparison of OncoTrace‐TOO, TOD‐CUP, and CUP‐AI‐Dx on TCGA validation dataset.

Model	Accuracy (%)
**OncoTrace‐TOO**	**96.69**
TOD‐CUP^a^	83.04
CUP‐AI‐Dx	80.92

*Note:* Bold values indicate the highest performance across all evaluated methods.

^a^ TOD‐CUP: Feature selection by median absolute deviation (MAD) and weighted ensemble k‐TSP algorithm for multiclass classification.

**FIGURE 5:**
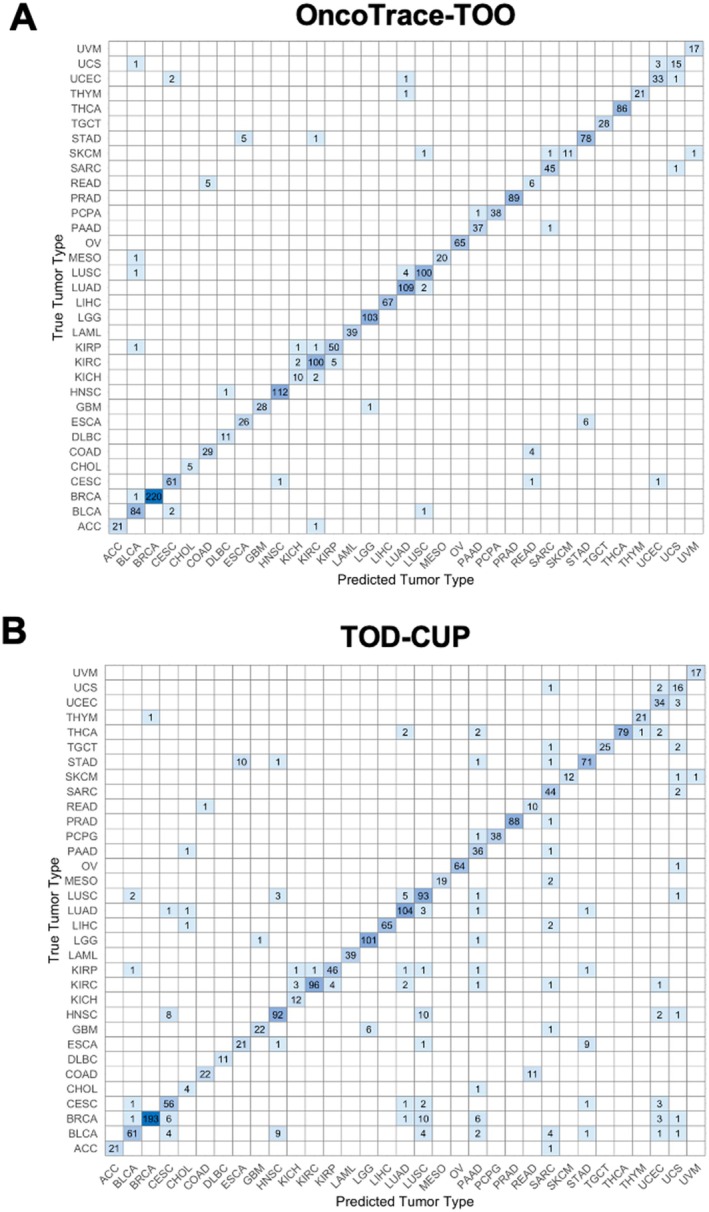
Comparison of multiclass classification results for 33 cancer types using the (A) OncoTrace‐TOO and (B) TOD‐CUP algorithms based on TCGA RNA‐seq data: confusion matrix showing multiclass classification performance across 33 cancer types.

Misclassifications primarily occurred between histologically related cancers, such as KIRC/KIRP (renal) and COAD/READ (gastrointestinal), underscoring shared molecular profiles (Figure 5). These errors reflect intrinsic biological similarities: for example, COAD and READ, both gastrointestinal cancers with overlapping histological and clinical features, exhibited near‐identical gene expression patterns at the molecular level. Similarly, misclassifications between ESCA and STAD (esophageal vs. gastric adenocarcinoma) align with their shared embryological origin and therapeutic paradigms.

Notably, the model's tendency to group misclassified samples within organ systems—such as renal (KIRC/KIRP/KICH), gastrointestinal (COAD/READ), and gynecological cancers (UCEC/CESC)—retains clinical relevance. While these errors highlight limitations in resolving histologically ambiguous subtypes, they provide valuable diagnostic insights. Clinically, tumors misclassified within the same organ system often receive similar treatment regimens (e.g., platinum‐based therapies for gynecological cancers [25]), ensuring that predictions remain actionable even when imperfect. Thus, while supplementary evidence is required for definitive diagnosis, the model's misclassifications offer biologically and therapeutically coherent guidance.

### 3.3 External Validation and Metastatic Cancer Performance

External validation using GEO datasets further confirmed the model's robustness across diverse cancer types and metastatic samples. For metastatic cancers, OncoTrace‐TOO achieved 99.73% accuracy in Skin Cutaneous Melanoma (SKCM, *n* = 369), with only one misclassified sample attributed to molecular similarities between SKCM and Sarcoma (SARC). The model also demonstrated perfect accuracy (100%) in Thyroid Carcinoma (*n* = 8), highlighting its ability to generalize to rare and heterogeneous metastases.

However, performance varied for metastatic breast cancer (*n* = 10), where the model achieved 70% accuracy. This lower accuracy likely stems from the limited sample size and the high molecular heterogeneity inherent in breast cancer metastases, which often exhibit divergent expression profiles compared to primary tumors. Despite this, the model's ability to correctly classify 7 out of 10 cases—including samples from both TCGA and GEO—underscores its potential utility in clinical scenarios where metastatic origins are ambiguous. Collectively, these results validate OncoTrace‐TOO's robustness in addressing the complexities of metastatic cancer classification while emphasizing the need for larger, metastasis‐specific training datasets to further refine performance (Table 4).

**TABLE 4:** Performance of OncoTrace‐TOO on external validation datasets and metastatic cancers.

Cancer	No. samples	Accuracy (%)	Primary/metastatic
Bladder cancer	12	100	Primary
Liver cancer	13	100	Primary
Clear cell renal cell carcinoma	47	100	Primary
Breast cancer	2	100	Primary
Skin cutaneous melanoma	369	99.73	Metastatic
Thyroid carcinoma	8	100	Metastatic
Breast cancer	10	70	Metastatic

### 3.4 Application of OncoTrace‐TOO to Clinical Cancer Samples

To preliminarily evaluate the applicability of our model to clinical samples, we established two independent clinical validation cohorts comprising 14 FFPE specimens spanning six tumor types (breast carcinoma, lung cancer, thyroid carcinoma, gastric adenocarcinoma, colorectal adenocarcinoma, and sarcoma). When applied to these external cohorts, the TCGA‐trained OncoTrace‐TOO model demonstrated clinical‐grade performance with 85.71% positive predictive value (Table 5), despite inherent biological complexities. Sarcomas—a diagnostically challenging entity due to histopathological overlap—served as a critical test case: among four molecularly distinct subtypes (Ewing sarcoma, synovial sarcoma, dedifferentiated liposarcoma [DDLS], and alveolar rhabdomyosarcoma), the model achieved 75% subtype‐specific accuracy. The single misclassification event (DDLS misidentified as bladder cancer) likely reflects shared molecular pathways in adipocytic differentiation. These results underscore the potential of OncoTrace‐TOO for single‐sample classification in clinical settings, highlighting its broad applicability and robustness across diverse tumor types.

**TABLE 5:** Clinical samples' validation of the OncoTrace‐TOO based on RNA‐seq data sets.

ID	Clinical diagnosis description	Prediction cancer type	Results
Patient 1	Breast invasive ductal carcinoma	Breast invasive carcinoma	P
Patient 2	Breast cancer	Breast invasive carcinoma	P
Patient 3	Breast invasive ductal carcinoma	Breast invasive carcinoma	P
Patient 4	Right breast malignant tumor	Breast invasive carcinoma	P
Patient 5	Lung cancer	Lung squamous cell carcinoma	P
Patient 6	Lung space‐occupying lesion	Lung adenocarcinoma	P
Patient 7	Multiple metastases of lung cancer	Lung squamous cell carcinoma	P
Patient 8	Rectal cancer	Stomach adenocarcinoma	M
Patient 9	Gastric cancer	Stomach adenocarcinoma	P
Patient 10	Ewing's sarcoma	Sarcoma	P
Patient 11	Dedifferentiated liposarcoma	Bladder urothelial carcinoma	M
Patient 12	Alveolar rhabdomyosarcoma	Sarcoma	P
Patient 13	Synovial sarcoma	Sarcoma	P
Patient 14	Thyroid cancer	Thyroid carcinoma	P

*Note:* P (Positive Match): Prediction matches the clinical diagnosis; M (Mismatch): Prediction does not match the clinical diagnosis.

### 3.5 Model Interpretability and Biological Insights

OncoTrace‐TOO algorithm demonstrates significant advantages in certain cancer types that are prone to misclassification. To elucidate the molecular drivers of its predictions, we employed Shapley values to quantify feature importance, accounting for the non‐linear relationships between genes and model outputs. This analysis identified the top 10 most influential genes for each of the 33 cancer types (Table S2), revealing both potential pan‐cancer biomarkers and subtype‐specific drivers.

**FIGURE 6:**
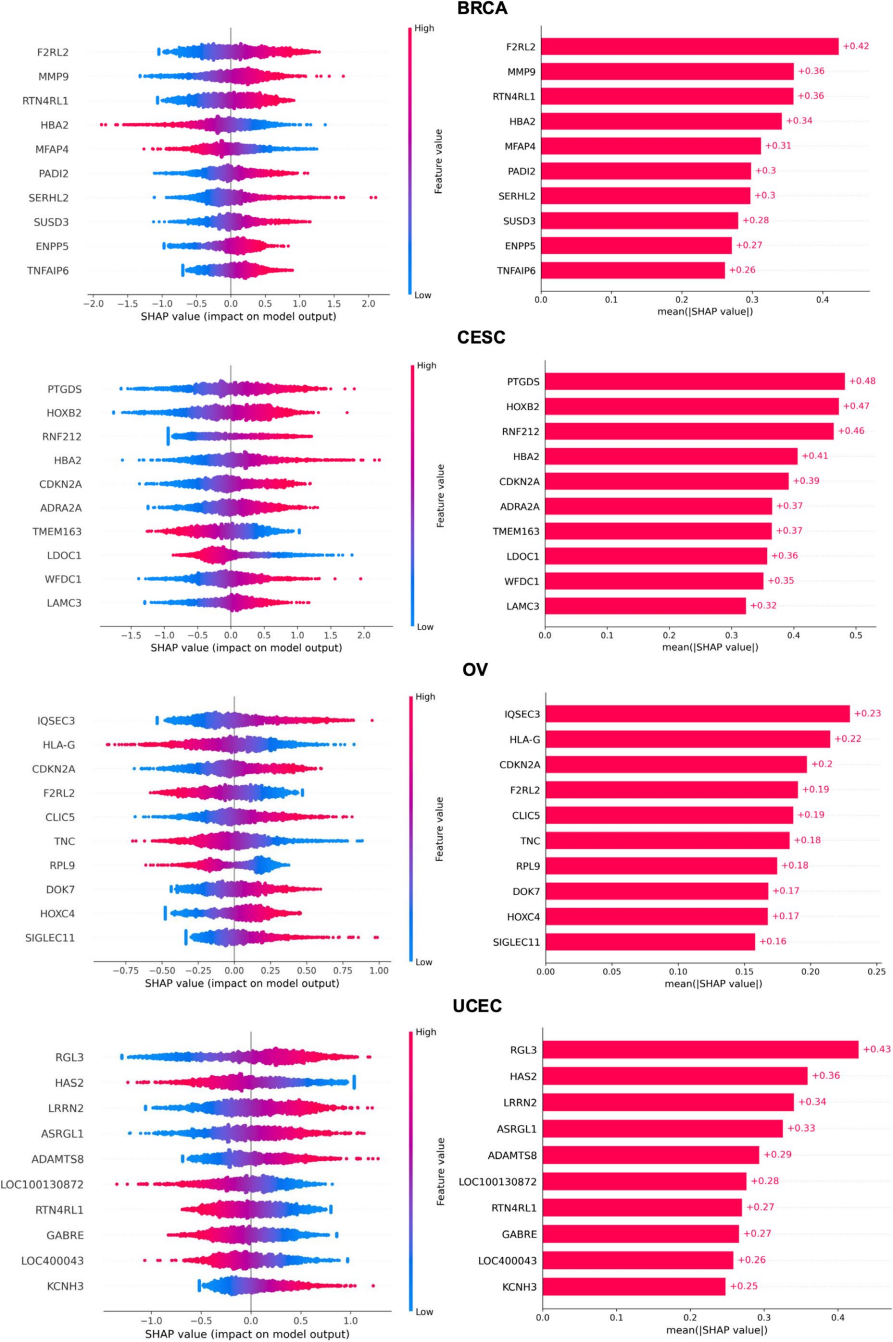
Individual type SHAP values for pan‐gynecologic cancers. The top 10 most important features contributing to accurate predictions of cancer types are included.

**FIGURE 7:**
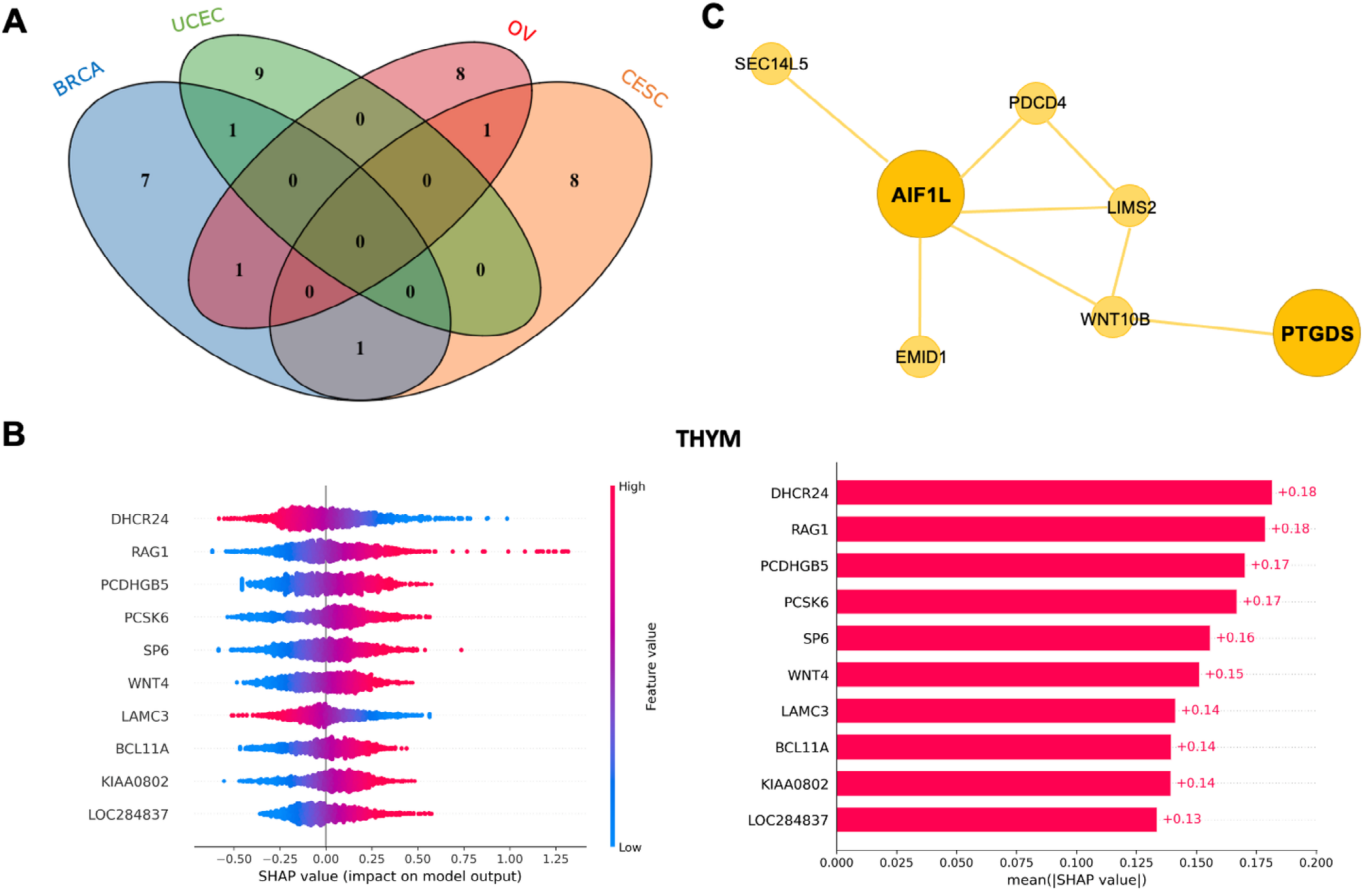
(A) Venn diagram of top 10 features for pan‐gynecologic cancers. (B) Top 10 features selected by SHAP values for Thymoma. (C) Sub‐network graph related to important genes in CHOL.

Focusing on gynecological cancers (BRCA, CESC, OV, and UCEC), a Venn diagram of the top 10 genes revealed subtype‐specific signatures (Figure 6). While only 1–2 genes (e.g., F2RL2) were shared across multiple cancers, their roles varied with the tumor microenvironment (Figure 7A). For example, F2RL2 was identified as a prominent gene in both breast cancer [26, 27] and ovarian cancer. In breast cancer, F2RL2 promotes tumor growth and metastasis by activating the thrombin signaling pathway, whereas in ovarian cancer, it accelerates tumor progression by modulating immune evasion mechanisms, such as modulating immune cell infiltration [28].

For under‐researched cancers like thymic carcinoma, the model prioritized DHCR24 and RAG1 (Figure 7B). DHCR24 promotes tumor proliferation, apoptosis resistance, and invasion through cholesterol biosynthesis [29], and activates the PI3K‐AKT signaling pathway [30, 31], while RAG1, reexpressed by tumor‐infiltrating T cells—may facilitate immune evasion via TCR specificity remodeling [32].

Notably, OncoTrace‐TOO achieved perfect accuracy in cancer types with limited training samples like cholangiocarcinoma (CHOL, *n* = 47). SHAP analysis identified PTGDS and AIF1L as top predictors (Table S2). PTGDS suppresses tumor growth via Wnt/β‐catenin modulation [33, 34, 35], while AIF1L plays a key role in regulating the cytoskeleton in breast cancer, contributing to cellular structure and function [36]. Subnetwork analysis revealed their interplay with WNT10B (Figure 7C), linking lipid metabolism, inflammation, and immune responses to CHOL pathogenesis [37]. Moreover, clinically significant therapeutic targets such as CD22 were also identified (Table S2). These results underscore OncoTrace‐TOO's ability to extract biologically meaningful associations even with sparse data, validating its utility for rare CUP diagnosis.

## 4 Discussion

Cancer of unknown primary (CUP) is a clinically challenging diagnosis, where metastatic cancers cannot be traced back to a primary tumor site. The diagnosis and treatment of CUP are often hindered by the lack of reliable molecular signatures, leaving physicians with limited information about the cancer's origin. In this study, we developed the OncoTrace‐TOO algorithm, which provides a more accurate and robust method for predicting the tissue of origin using protein‐coding mRNA expression data. OncoTrace‐TOO, which predicts both primary and metastatic cancer types accurately even with limited training data, underscores its broad applicability and potential for generalization across diverse cancer types.

Unlike prior methods such as TOD‐CUP, which collapse tumors into 24 broader organ systems, OncoTrace‐TOO retains the full granularity of 33 TCGA cancer types. This fine‐grained classification enables the model to differentiate between biologically and anatomically similar tumors, such as colon versus rectal adenocarcinomas, or lung squamous versus head and neck squamous carcinomas. Importantly, it also reduces misclassification among cancer types with shared embryologic origins or overlapping transcriptomic profiles—such as kidney cancers (KIRC/KIRP/KICH) and gastrointestinal cancers (COAD/READ/STAD)—indicating superior resolution of closely related subtypes. While CUP‐AI‐Dx employs a sophisticated deep learning architecture, its performance shows substantial variability across cancer types. This may be attributed to its reliance on 817 transcriptomic features, which, in the limited samples for some cancers, may increase susceptibility to overfitting and reduce generalizability.

By incorporating Shapley value‐based feature importance analysis, we gained deeper insights into the molecular mechanisms driving the classification process. This revealed tumor‐specific potential markers, several of which have been previously implicated in tumor biology (e.g., *F2RL2* in gynecological cancers, *DHCR24* and *RAG1* in thymic carcinoma, *PTGDS* in cholangiocarcinoma). These findings lend support to the biological plausibility of the model's predictions and suggest that OncoTrace‐TOO may capture features associated with oncogenic processes, rather than reflecting tissue identity alone.

Although CUP tumors were not directly included in this study, previous research has shown that latent primary CUP, where the primary site becomes known over time, can be predicted with accuracy comparable to metastatic tumors of known origin [2]. Nevertheless, substantial challenges exist in validating TOO classifiers in CUP. First, the absence of a definitive gold standard in CUP diagnosis makes it difficult to rigorously validate classifier performance. While some CUP cases may be retrospectively resolved through latent primary detection or autopsy, such instances are limited in number and prone to selection or diagnostic biases. Furthermore, CUP tumors may exhibit molecular divergence from their presumed primaries due to clonal evolution, treatment exposure, or atypical metastatic routes, which can obscure transcriptomic features used for classification [38].

In addition to biological differences, tissue scarcity and compromised RNA quality—especially in archived or minimally invasive specimens—pose additional barriers to the implementation of transcriptome‐based classifiers. While OncoTrace‐TOO was trained using bulk RNA‐seq data from fresh‐frozen tissues, its architecture is based on protein‐coding genes and rank‐based expression features, which may lend some robustness to lower‐input or partially degraded samples. With appropriate re‐training and validation, the model could potentially be adapted for use in more clinically accessible formats, such as targeted RNA panels, FFPE‐derived transcriptomes, or even cell‐free RNA (cfRNA) profiles [39].

Despite our model performing well in identifying the tissue of origin for most cancers, it is also important to recognize that the clinical benefit of accurate TOO classification in CUP remains under investigation. While origin prediction is a critical first step, previous studies [40, 41] have not consistently demonstrated improved survival with site‐specific therapy guided by molecular assays. To address this, future work should incorporate predictive biomarkers, drug response signatures, and pathway activity profiles, enabling not only accurate classification but also actionable guidance for precision therapy. Additionally, the current framework is built exclusively on transcriptomic data, which may not fully capture the molecular heterogeneity observed in poorly differentiated or highly metastatic tumors. Emerging evidence [42, 43] have demonstrated that enhancing the model's capabilities by integrating genomics data (e.g., DNA methylation, mutations, and copy number variation) can enrich the feature space, particularly in difficult‐to‐classify and poorly differentiated tumors. Given the high heterogeneity observed in metastatic tumors, particularly in cancers like colorectal carcinoma [44], gastric cancer [45] and hepatocellular carcinoma [46], improving classification accuracy may require integrating spatial, epigenomic, or multi‐omics features to capture their complexity [47, 48]. Moreover, our model successfully identifies important genes across different cancer types, undergoing its potential to uncover novel therapeutic targets or diagnostic biomarkers. Future prospective evaluation of OncoTrace‐TOO in larger, diverse, and independently collected CUP cohorts will be essential to determine its generalizability and clinical readiness.

In conclusion, this study introduces a novel TOO classification method that provides accurate predictions and insights into the molecular mechanisms driving cancer, offering potential advancements in metastatic cancer diagnosis and treatment, particularly for managing cancers with unknown primary origins.

## Author Contributions

**Jianquan Zhang**, **Yang Hao:** conceptualization. **Yang Hao**, **Haochun Huang:** methodology. **Yang Hao:** validation. **Yang Hao**, **Haochun Huang**, **Daiyun Huang:** formal analysis. **Yang Hao:** data curation. **Yang Hao:** writing – original draft preparation. **Daiyun Huang**, **Xin Liu**, **Jianquan Zhang:** writing – review and editing. **Jianwen Ruan:** visualization. All authors have read and agreed to the published version of the manuscript.

## Conflicts of Interest

The authors declare no conflicts of interest.

## Supporting information


**Table S1:** Performance with different classifiers (k = 200).
**Table S2:**. Top 10 genes ranked by SHAP values across 33 cancer types.

## Data Availability

The data that support the findings of this study are available on request from the corresponding author. The data are not publicly available due to privacy or ethical restrictions. The OncoTrace‐TOO dataset generation and model codes are available at https://github.com/XiaoLangJun666/OncoTrace‐TOO.
